# Characterization of *Plasmodium falciparum* ATP-dependent DNA helicase RuvB3

**DOI:** 10.1186/s12936-016-1573-2

**Published:** 2016-11-03

**Authors:** Paviga Limudomporn, Saengduen Moonsom, Ubolsree Leartsakulpanich, Pattra Suntornthiticharoen, Songsak Petmitr, Michael Weinfeld, Porntip Chavalitshewinkoon-Petmitr

**Affiliations:** 1 Department of Protozoology, Faculty of Tropical Medicine, Mahidol University, 420/6 Rajvithi Road, Bangkok, 10400 Thailand; 2 National Center for Genetic Engineering and Biotechnology, National Science and Technology Development Agency, 113 Thailand Science Park, Pahonyothin Rd, Pathumthani, 12120 Thailand; 3 Department of Biomedical Sciences, Faculty of Science, Rangsit University, Lak Hok, Pathumthani, 12000 Thailand; 4 Department of Molecular Tropical Medicine and Genetics, Faculty of Tropical Medicine, Mahidol University, Bangkok, 10400 Thailand; 5 Department of Oncology, Cross Cancer Institute, University of Alberta, Edmonton, AB T6G 1Z2 Canada

**Keywords:** *Plasmodium falciparum*, ATP-dependent DNA helicase, ATPase activity, Helicase activity, PfRuvB3

## Abstract

**Background:**

Malaria is one of the most serious and widespread parasitic diseases affecting humans. Because of the spread of resistance in both parasites and the mosquito vectors to anti-malarial drugs and insecticides, controlling the spread of malaria is becoming difficult. Thus, identifying new drug targets is urgently needed. Helicases play key roles in a wide range of cellular activities involving DNA and RNA transactions, making them attractive anti-malarial drug targets.

**Methods:**

ATP-dependent DNA helicase gene (*PfRuvB3*) of *Plasmodium falciparum* strain K1, a chloroquine and pyrimethamine-resistant strain, was inserted into pQE-TriSystem His-Strep 2 vector, heterologously expressed and affinity purified. Identity of recombinant PfRuvB3 was confirmed by western blotting coupled with tandem mass spectrometry. Helicase and ATPase activities were characterized as well as co-factors required for optimal function.

**Results:**

Recombinant PfRuvB3 has molecular size of 59 kDa, showing both DNA helicase and ATPase activities. Its helicase activity is dependent on divalent cations (Cu^2+^, Mg^2+^, Ni^+2^ or Zn^+2^) and ATP or dATP but is inhibited by high NaCl concentration (>100 mM). PfPuvB3 is unable to act on blunt-ended duplex DNA, but manifests ATPase activity in the presence of either single- or double-stranded DNA. PfRuvB3.is inhibited by doxorubicin, daunorubicin and netropsin, known DNA helicase inhibitors.

**Conclusions:**

Purified recombinant PfRuvB3 contains both DNA helicase and ATPase activities. Differences in properties of RuvB between the malaria parasite obtained from the study and human host provide an avenue leading to the development of novel drugs targeting specifically the malaria form of RuvB family of DNA helicases.

## Background

Malaria is one of the most serious and widespread parasitic diseases of humans. Globally, in 2015 an estimated 3.2 billion people were at risk of being infected with malaria parasites and contracting the disease, with approximately 214 million people becoming infected resulting in a mortality of 438,000 [1]. With the emergence of drug-resistant parasites to all available anti-malarials, control of malaria is becoming difficult [1, 2]. This has led to efforts in developing novel strategies and in a search for new drug targets with which to combat the scourge of malaria.

The availability of the complete genome sequence of *Plasmodium falciparum*, the causative agent of fatal malaria, has opened new avenues to identify genes important for parasite survival. Many potential chemotherapeutic targets involved in various metabolic pathways at different malaria parasite life stages have been identified recently [3]. Among these, helicases constitute a highly conserved group of enzymes important in all aspects of nucleic acid metabolism, such as replication, recombination, repair, transcription and (RNA) stability [3–6].

RuvB is an ATP-dependent DNA helicase with a hexameric ring structure and its architecture has been suggested to be related to those of members of ATPases associated with various cellular activities (AAA+) protein class [6]. In *Escherichia coli*, RuvAB helicase is an ATP-driven translocase that promotes branch migration of Holliday junction and formation of heteroduplex DNA [6], in addition to being essential for replication fork reversal at occurrences of DNA replication defects [7]. Expression of *E. coli* ruvB genes is required for DNA repair and maintaining normal levels of cellular resistance to stress-induced mutagenesis, so their resulting mutants are thereby defective in recombination [8, 9]. In eukaryotes, RuvB proteins, such as yeast RvB1 and RvB2, are nuclear proteins indispensable for cell cycle progression and RNA polymerase II-dependent transcription [10]. In addition, RuvBs are involved directly in the regulation of transcription of over 5% of yeast genes as essential components of a chromatin remodelling complex determining genes regulated by the complex [11] and indirectly by recruiting the TATA-binding protein (TBP) to the promoter and its impairment confer growth defects [12], and they are essential for viability in all model organisms including *Saccharomyces cerevisiae* [13], *Drosophila melanogaster* [14] and *Caenorhabditis elegans* [15]. Mutations in the conserved ATP-binding and hydrolysis motifs of RuvBs decrease viability of these organisms [11].

Given that RuvBs play essential roles in nearly all aspects of nucleic acid metabolism, *P. falciparum* RuvBs should be no exception. Analysis of *P. falciparum* genome database identifies at least three *RuvB* homologues, namely, *PfRuvB1, PfRuvB2 and PfRuvB3* [16]. *PfRuvB3*, located on chromosome 13, comprises of 1452 bp encoding for a 483-amino acid protein [17]. Recombinant PfRuvB1 and PfRuvB2 containing both helicase and ATPase activities, and have been heterologously produced in *E. coli* and their properties characterized [18, 19]. However, recombinant PfRuvB3 shows only ATPase activity, unlike that of the purified parasite protein that contains both helicase and ATPase activities [19, 20]. It is possible that the reported conditions for cloning and expression were not optimal for producing fully active enzyme. Here, conditions for generating PfRuvB3 dual activity in *E. coli* were examined and the effects of a number of known helicase inhibitors on the recombinant enzyme were also evaluated.

## Methods

### Cloning of *Plasmodium falciparum* ATP-dependent DNA helicase gene (*PfRuvB3*)

Full length *PfRuvB3* from *P. falciparum* K1, a chloroquine and pyrimethamine-resistant strain from Thailand [21], was PCR amplified using primers 5′-TCCCCCGGGGCATGAAGCTCGAAGAAG-3′ (with a *Sma*I site (underlined) upstream of the start codon) and 5′-CCGCTCGAGATTACTTGTACTTGAATTTTCCG-3′ (with a *Xho*I site shown underlined) based on genome sequence of *P. falciparum* 3D7 (NCBI database accession no. XM001350297.1). Thermocycling conditions were as follows: 95 °C for 5 min; followed by 35 cycles of 95 °C for 10 s, 61.5 °C for 30 s and 72 °C for 1 min; with a final step at 72 °C for 90 s. The PCR was carried out using Phusion^®^ High-Fidelity DNA Polymerase (Thermo Scientific, MA, USA) and amplicon was purified using Nucleospin^®^ extract II kit (Macherey-Magel, Düren, Germany), digested with *Sma*I and *Xho*I, and then ligated to similarly digested pQE-TriSystem His-Strep 2 expression vector (Qiagen, Germany) using T4 DNA ligase. The presence of *PfRuvB3* in the recombinant plasmid was further verified for its nucleotide sequence by BioDesign (Pathumthani, Thailand).

### Heterologous expression of *PfRuvB3* and purification of recombinant protein

Recombinant pQE-TriSystem His-Strep 2-*PfRuvB3* plasmid was transfected into *Mix & Go* Competent Cells-Strain JM109 (Zymo Research Corporation, CA, USA) and transformed cells were selected on LB agar containing 100 μg/ml of ampicillin at 37 °C overnight. Cultures were inoculated into LB broth containing 100 μg/ml of ampicillin and grown at 37 °C until A_600 nm_ reached 0.4-0.6, and then treated with 1 mM isopropyl β-d-1-thiogalactopyranoside (IPTG) at 37 °C for 1 h with shaking. Cells were harvested by centrifugation at 2000×*g* at 4 °C for 15 min, washed with buffer (50 mM NaH_2_PO_4_ pH 8.0, 300 mM NaCl and 10 mM imidazole) and re-suspended in buffer supplemented with protease inhibitor cocktail (complete™ ULTRA Tablets, Roche, Germany) before being lysed by sonication. Following centrifugation at 9500×*g* at 4 °C for 45 min, supernatant was applied onto a Ni–NTA affinity column (Qiagen, Hilden, Germany), which was washed with washing buffer (50 mM NaH_2_PO_4_ pH 8.0, 300 mM NaCl, 20 mM imidazole and 1 mM PMSF) until no protein was detected in washed fractions. Recombinant PfRuvB3 was eluted with elution buffer (50 mM NaH_2_PO_4_ pH 8.0, 300 mM NaCl and 250 mM imidazole) and fractions collected were subjected to analysis SDS-PAGE. Fractions containing the 6xHistidine-tag fusion protein were pooled and applied onto a 1 ml *Strep*-Tactin^®^ Sepharose^®^ column (Iba Life Sciences, Germany), which was washed with 5 ml of buffer A (100 mM Tris–HCl pH 8.0, 150 mM NaCl and 1 mM EDTA) and recombinant PfRuvB3 was eluted with buffer A containing 2.5 mM desthiobiotin. The recombinant protein was concentrated using an Amicon^®^ Ultra-15 filtration unit and stored in buffer B (50 mM NaH_2_PO_4_ pH 8.0, 100 mM NaCl and 30% glycerol). Protein concentration was measured using Bradford assay (Bio-Rad, CA, USA) and protein purity was checked by SDS-PAGE.

### Western blot and tandem mass spectrometry analysis

Following SDS-PAGE protein was transferred onto nitrocellulose membrane (Bio-Rad), which then was incubated with phosphate-buffered saline (PBS) containing 3% (w/v) bovine serum albumin (BSA) and 0.5% (v/v) Tween 20 for 1 h at room temperature, washed three times with PBS buffer containing 0.1% (v/v) Tween 20 and incubated with *Strep*-Tactin^®^ horse radish peroxidase conjugate (diluted 1:100 in PBS/BSA/Tween buffer) for 1 h at room temperature. After washing 2 times, protein was visualized using SuperSignal™ West Pico Chemiluminescent Substrate (Thermo Scientific). Protein band stained with Coomassie blue dye was eluted from SDS–polyacrylamide gel, digested with trypsin and amino acid sequence of the peptides analyzed using Synapt HDMS Q-TOF LC–MS/MS equipped with MASCOT software (Waters, UK).

### Preparation of DNA helicase substrates

DNA helicase substrates (Table 1) were prepared as follows. Oligodeoxynucleotides were 5′-end labelled using T4 polynucleotide kinase and [γ-^32^P] ATP (800 CimMol^−1^; PerkinElmer, MA, USA). Partial duplex of either short or long oligonucleotide was produced by annealing 34 μM [^32^P]-labelled oligodeoxynucleotide type 1 or 4 (Table 1) to single-stranded circular M13mp18 (+) DNA (New England Biolabs, MA, USA) in 20 mM Tris–HCl pH 7.5 containing 10 mM MgCl_2_, 100 mM NaCl and 1 mM DTT, incubating at 95 °C for 5 min and then cooling down slowly to room temperature over a period of 1 h [22]. Blunt ended DNA duplex was constructed by annealing 5 ng of 5′ end [^32^P]-labelled oligonucleotide type 5–50 ng of oligodeoxynucleotide type 6 in 40 mM Tris–HCl pH 7.5 containing 20 mM MgCl_2_, and 50 mM NaCl at 95 °C for 5 min and then cooling to room temperature as described above. Fork-like substrates with 3′, 5′ or both 3′ and 5′ overhanging ends were generated by annealing single-stranded circular M13mp18 (+) DNA with 5′ end [^32^P]-labelled oligodeoxynucleotide type 2, 3 and 7, respectively.

**Table 1:** Oligonucleotides and DNA duplex substrates used in the study

Oligonucleotide type	Sequence: 5′-3′
1. 17mer	5′-GTAAAACGACGGCCAGT-3′
2. 32mer-I	5′-TTTTTTTTTTTTTTTGTTTTCCCAGTCACGAC-3′
3. 32mer-II	5′-GTTTTCCCAGTCACGACTTTTTTTTTTTTTTT-3′
4. 34mer	5′-ATAAAAATTTTTAGAACCCTCATATATTTTAAAT-3′
5. 41mer-I	5′-AATTCGAGCTCGGTACCCGGGGATCCTCTAGAGTCGACCTG-3′
6. 41mer-II	5′-CAGGTCGACTCTAGAGGATCCCCGGGTACCGAGCTCGAATT-3′
7. 47mer	5′-TTTTTTTTTTTTTTTGTTTTCCCAGTCACGACTTTTTTTTTTTTTTT-3′

DNA duplex	Component
Short oligonucleotide	M13mp18 + 17mer
Long oligonucleotide	M13mp18 + 34 mer
Blunted end	41 mer-I + 41 mer-II
3′overhang	M13mp18 + 32 mer-II
5′overhang	M13mp18 + 32 mer-I
3′and 5′ overhang	M13mp18 + 47 mer

### DNA helicase activity assay

Unwinding of [^32^P]-labelled partial duplex DNA was employed as a measurement of helicase activity. The reaction mixture (10 µl) contained 20 mM Tris–HCl pH 9.0, 8 mM DTT, 2 mM MgCl_2_, 2 mM ATP, 10 mM KCl, 4% (w/v) sucrose, 80 mg/ml BSA, [^32^P]-labelled DNA duplex substrate (Table 1) and purified PfRuvB3. The assay was performed at 37 °C for 90 min (unless indicated otherwise) and terminated with addition of 10X DNA loading dye (Fermentas, USA) and further incubation at 37 °C for 5 min. Helicase substrate and product were separated by 12% non-denaturing PAGE and then the gel was exposed to an X-ray film. DNA unwinding was quantified by densitometric analysis of exposed substrate and product bands using GeneTools (Syngene). Percent unwinding is calculated using the following formula: [band density of product [single-stranded (ss)DNA] / (band density of ssDNA + band density of substrate (duplex DNA)] × 100 minus [percent unwinding in the absence of enzyme].

### Requirement of co-factors and effects of other factors

The ability of PfRuvB3 to hydrolyze different NTPs was studied by including 2 mM dATP, dCTP, dGTP, or dTTP in place of ATP in the standard reaction assay. The effects of ATP concentrations ranging from 0.3 to 20 mM on helicase activity were also determined. Divalent cation requirement was investigated mixture 2 mM CaCl_2_, CuCl_2_, NiSO_4_, ZnCl_2_, FeSO_4_, MnCl_2_ or MgSO_4_ in place of MgCl_2_. Effects of salt (0-200 mM NaCl in place of 10 mM KCl) and EDTA (5 mM) also were evaluated.

### Effect of DNA helicase inhibitors on PfRuvB3 activity

Known DNA helicase inhibitors, aphidicolin genistein, daunorubicin, doxorubicin, mitoxantrone, and netropsin (Sigma, USA) were prepared as 10^−2^ M stock solutions in dimethylsulfoxide (DMSO) and stored at −20 °C until used. All compounds were diluted with 10 mM Tris–HCl, pH 9.0 to specified concentrations for the assay. M13-17-mer duplex DNA was pre-incubated with 0.1 to 50 μM drug for 10 min prior to addition to the standard helicase assay mixture. The 0.5% (v/v final concentration) of DMSO was used as solvent control to determine its effects on helicase activity. The dose–response curves of relative activities of PfRuvB3 and various drug concentrations were generated, and half-maximal inhibitory concentration (IC_50_), the concentration of compound to inhibit enzyme activity by 50%, was obtained using GraphPad Prism 6.01 software.

### PfRuvB3 ATPase activity assay

ATPase reaction mixture (10 μl), containing reaction buffer (20 mM Tris–HCl pH 8.0, 8 mM DTT, 2 mM MgCl_2_, 10 mM KCl, 4% (w/v) sucrose and 80 mg/ml BSA), 1 μg of recombinant PfRuvB3, 50 ng of M13 mp18 (New England Biolabs, USA) or short oligonucleotide duplex (Table 1), 1 mM ATP and 85 nM [α-^32^P] ATP (Perkin and Elmer, USA), was incubated at 37 °C for 90 min. The reaction was terminated by the addition of 2 µl of 100 mM EDTA. One µl aliquot of the reaction mixture was spotted onto a PEI cellulose thin layer chromatography plate (Sigma), which was developed in 2 M acetic acid containing 1.5 M LiCl_2_ to separate ADP and ATP. Plate was dried and exposed to X-ray film and exposed bands quantified by densitometry as described above. Percent of [α-^32^P] ATP hydrolyzed is calculated using the formula: (band density of ADP/band density of ADP + band density of ATP) ×100 minus (percent ATP hydrolyzed in the absence of enzyme). Effective helicase inhibitors were investigated for their inhibitory effects on PfRuvB3 ATPase activity.

## Results

### Production and purification of recombinant PfRuvB3

**Fig. 1:**
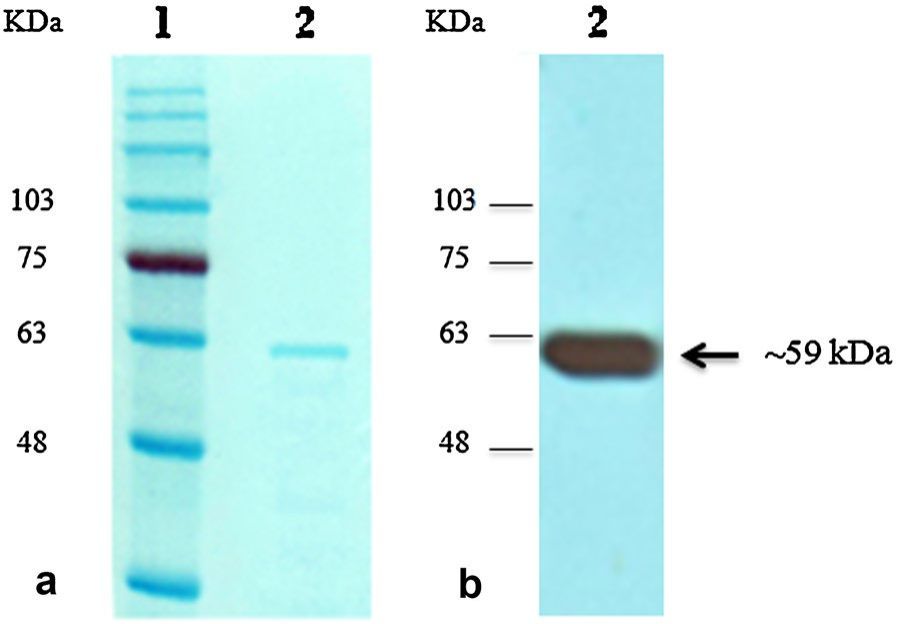
SDS-PAGE (**a**) and western blot (**b**) of purified PfRuvB3. Conditions for enzyme expression and purification are described under “Heterologous expression of PfRuvB3 and purification of recombinant protein”. The purified PfRuvB3 was analyzed by electrophoresis on a 12% SDS gel. Western blotting was performed using *Strep*-Tactin^®^ horse radish peroxidase conjugate. *Lane 1*, molecular weight markers (kDa); *lane 2*, purified recombinant PfRuvB3

Specific primers corresponding to ATP-dependent DNA helicase gene sequence of *P. falciparum* strain 3D7 were used to amplify a 1456-bp fragment from chloroquine and pyrimethamine-resistant *P. falciparum* K1 strain. A His-Strep-tagged PfRuvB3 protein with a molecular weight of 59 kDa was heterologously produced and affinity purified (Fig. 1a). Western blot analysis using *Strep*-Tactin^®^ conjugate detected the expected PfRuvB3 band (Fig. 1b). Based on amino acid sequence analysis, all 13 peptides obtained from mass spectrometry were matched with the sequence of *P. falciparum* 3D7 PfRuvB3 with ion scores of 4512, indicating an extensive homology (p value <0.05).

### PfRuvB3 DNA helicase activity

**Fig. 2:**
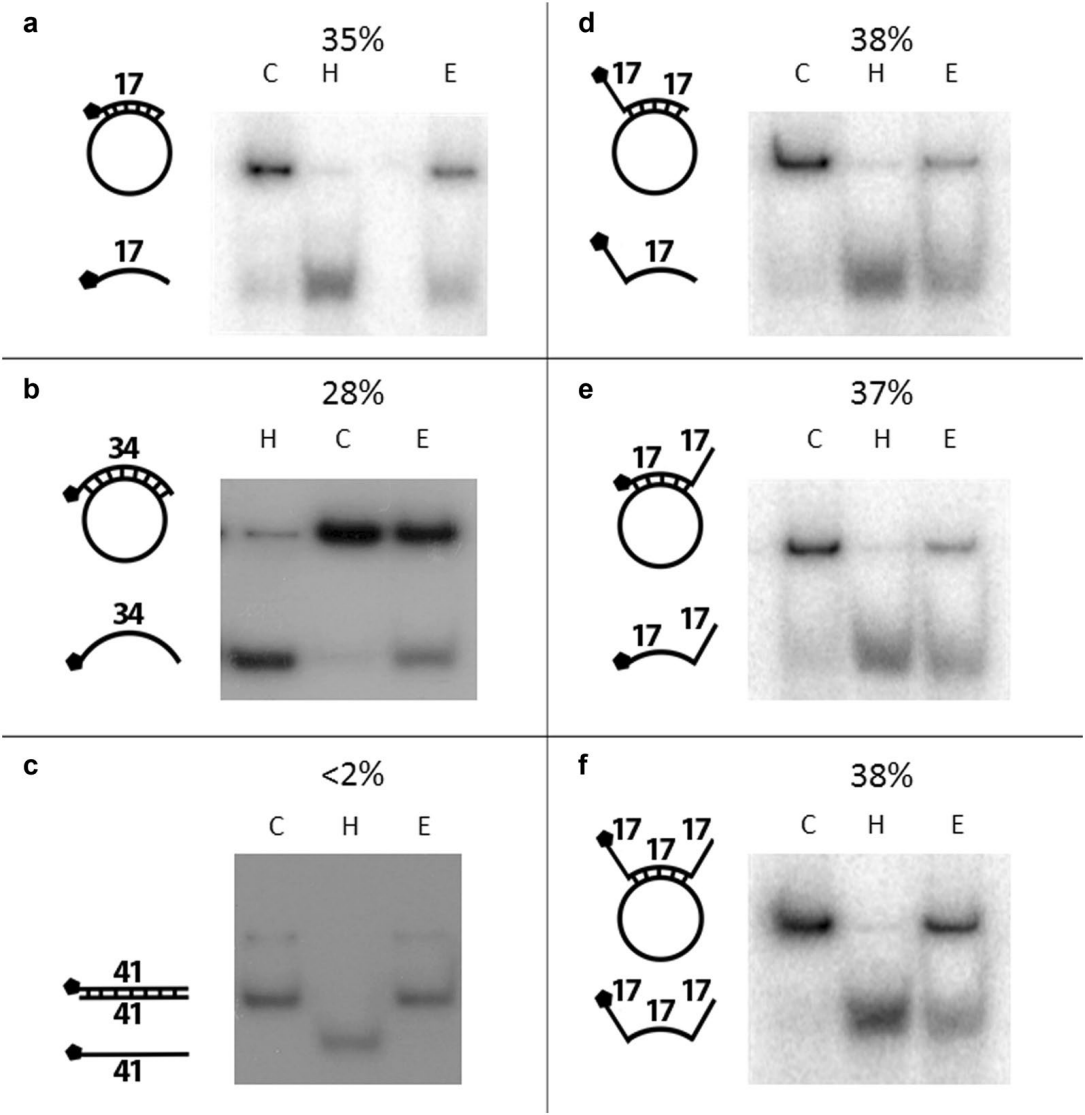
Substrate preference of recombinant PfRuvB3. Substrate preparation and assay conditions were as described under “Preparation of DNA helicase substrates” and “DNA helicase activity assay”. Helicase assays were carried out by using 1 μg of purified PfRuvB3 at 37 °C 90 min. Each* panel* shows substrate used and autoradiogram on gel. DNA duplex substrates in panels **a**, **b**, **c**, **d**, **e** and **f** were short oligonucleotide, long oligonucleotide, blunted end, 5′ overhang, 3′ overhang and 3′, 5′ overhang, respectively. Pentagon denotes ^32^P-labelled end. Percent unwinding is shown above each autoradiogram.* Lanes C* and* H* are control reaction without enzyme and heat-denatured substrate respectively.* Lane E* is reaction with recombinant PfRuvB3

A series of double stranded DNA substrates were prepared (Table 1) to examine the properties of PfRuvB3 helicase activity. PfRuvB3 was able to unwind short (17 mer), long (34 mer) and fork-like (32 and 47 mer) oligonucleotides present in duplex DNA with the same relative activity, but not blunt-ended duplex DNA (Fig. 2c).

**Fig. 3:**
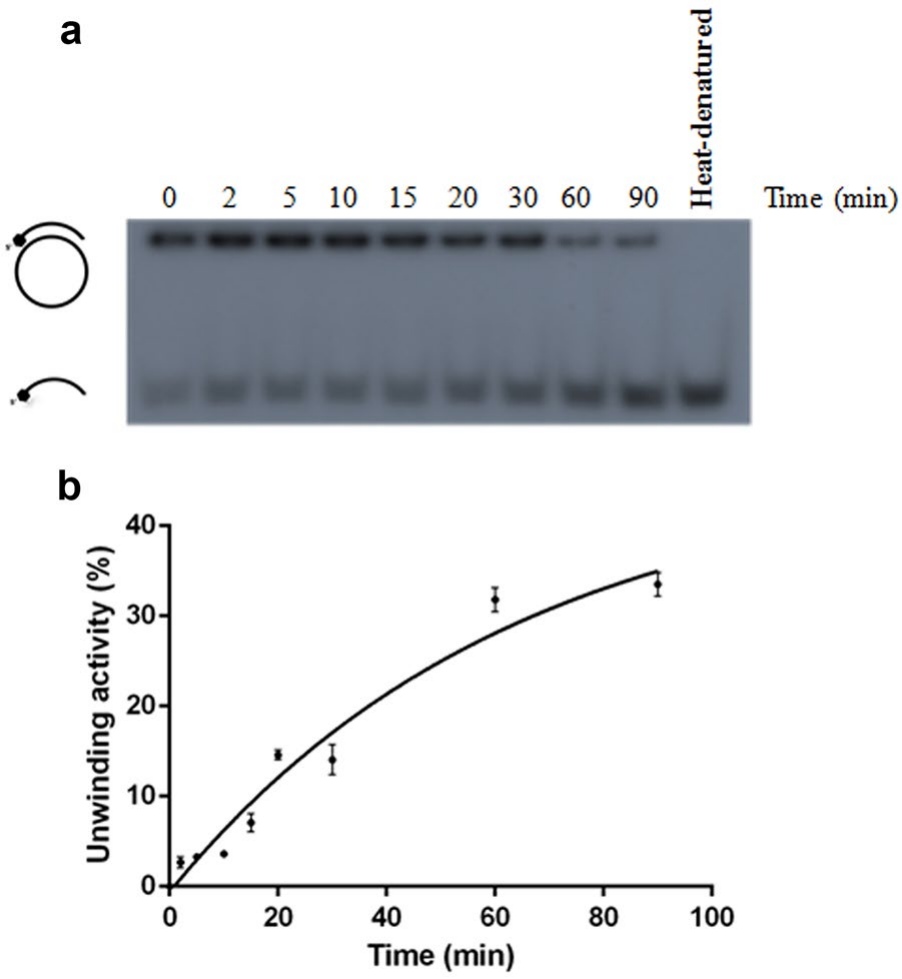
Time-dependent unwinding activity of recombinant PfRuvB3. Effects of incubation time on helicase activity of PfRuvB3 were determined using short oligonucleotide DNA duplex (Table 1) as a template. Assay conditions were as described under “DNA helicase activity assay”. Helicase assays were performed by using 1 μg of purified PfRuvB3 at 37 °C under various incubation periods as specified in the figure. **a** Autoradiogram. **b** Kinetics of unwinding activity of recombinant PfRuvB3. Result is shown as mean ± SD of three replicates

**Fig. 4:**
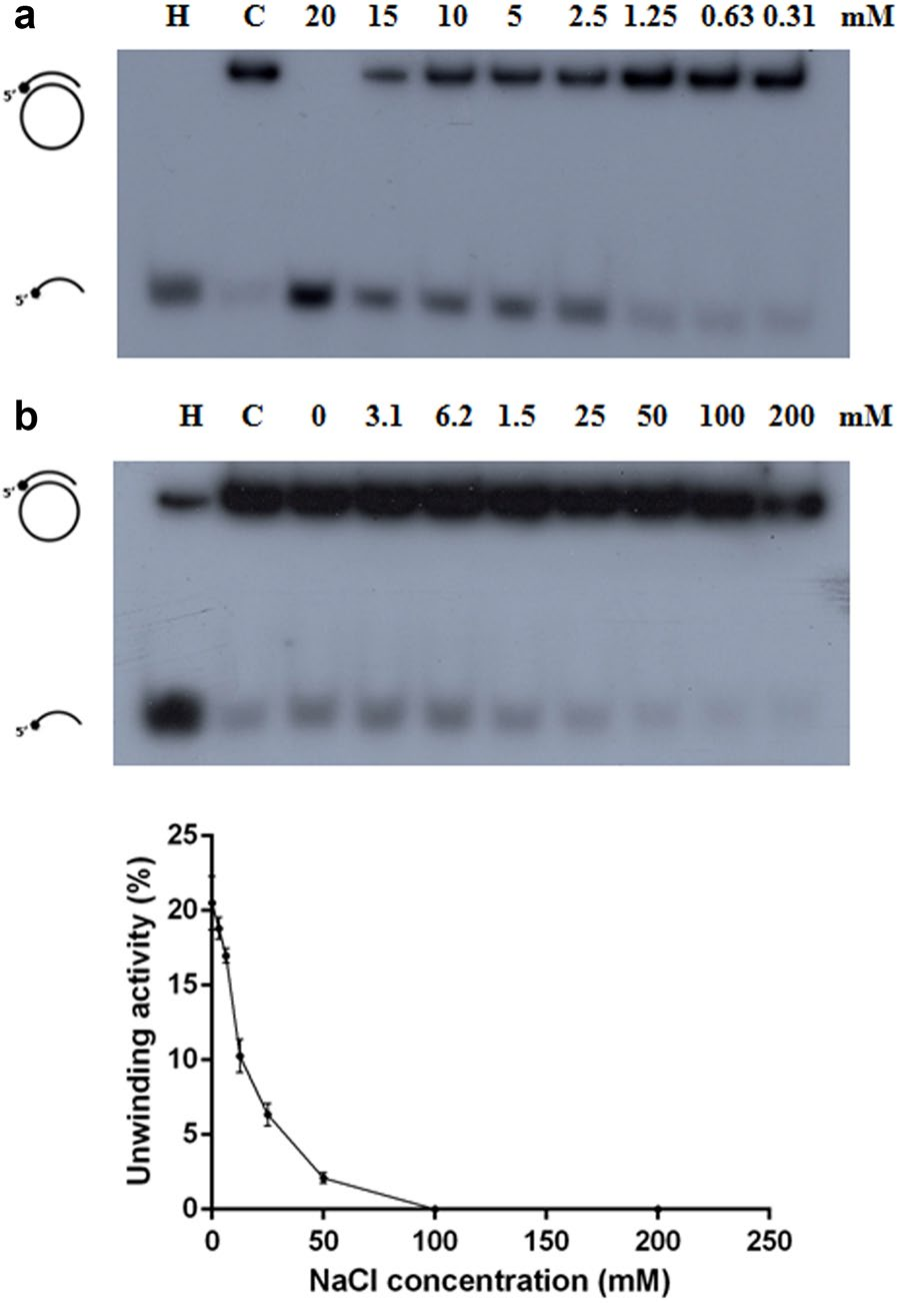
Effects of ATP (**a**) and NaCl (**b**) on unwinding activity of recombinant PfRuvB3. Their effects were investigated using short oligonucleotide DNA duplex (Table 1) as a template. Assay conditions were as described under “DNA helicase activity assay”. *Panel* shows autoradiogram. *Lanes C* and *H* are control reaction without enzyme and heat-denatured substrate respectively. Result is shown as mean ± SD of three replicates

PfRuvB3 displayed time- and ATP concentration-dependent DNA helicase activity using the short oligonucleotide DNA duplex substrate (Figs. 3 and 4a). The ATP is required for the unwinding activity and increasing activity can still be observed up to 20 mM ATP (Fig. 4a). Of other dNTPs (dATP, dCTP, dGTP, and dTTP) tested, only dATP supported DNA unwinding activity of PfRuvB3 with similar activity as ATP (Table 2).

**Table 2:** Requirement conditions of PfRuvB3 unwinding activity

Factor	Reaction condition	Relative activity (%)
	Standard reaction^a^	35
	−Enzyme	<2
dNTP	−ATP + dATP^b^	33
	−ATP + dCTP	<2
	−ATP + dTTP	<2
	−ATP + dGTP	<2
Divalent cation	−MgCl_2_ + CaCl_2_^c^	<2
	−MgCl_2_ + CuCl_2_	36
	−MgCl_2_ + NiCl_2_	34
	−MgCl_2_ + ZnCl_2_	30
	−MgCl_2_ + FeSO_4_	<2
	−MgCl_2_ + MnCl_2_	<2
	−MgCl_2_ + MgSO_4_	37
Salt	+NaCl (200 mM)	<2
	+EDTA (5 mM)	<2

^a^20 mM Tris–HCl (pH 9.0), 8 mM DTT, 2 mM MgCl_2_, 2 mM ATP, 10 mM KCl, 4% (w/v) sucrose, 80 mg/ml BSA, ^32^P-labelled helicase substrate and 1 μg of purified PfRuvB3

^b^2 mM

^c^2 mM

RuvB requires the presence of divalent cations as a cofactor for ATP hydrolysis. Mg^2+^ was necessary for PfRuvB3 unwinding activity and could be replaced with Cu^2+^, Ni^2+^, Co^2+^ and Zn^2+^, but not with Ca^2+^, Fe^2+^ or Mn^2+^ (Table 2). The absolute requirement of PfRuvB3 helicase activity for divalent cations was further confirmed by addition of the metal chelator EDTA (5 mM) into the reaction mixture, which resulted in complete loss of helicase function.

The unwinding activity of PfRuvB3 was inhibited with increase in NaCl concentration and was almost absent in the presence of 100 mM of NaCl (Fig. 4b).

### PfRuvB3 ATPase activity assay

**Fig. 5:**
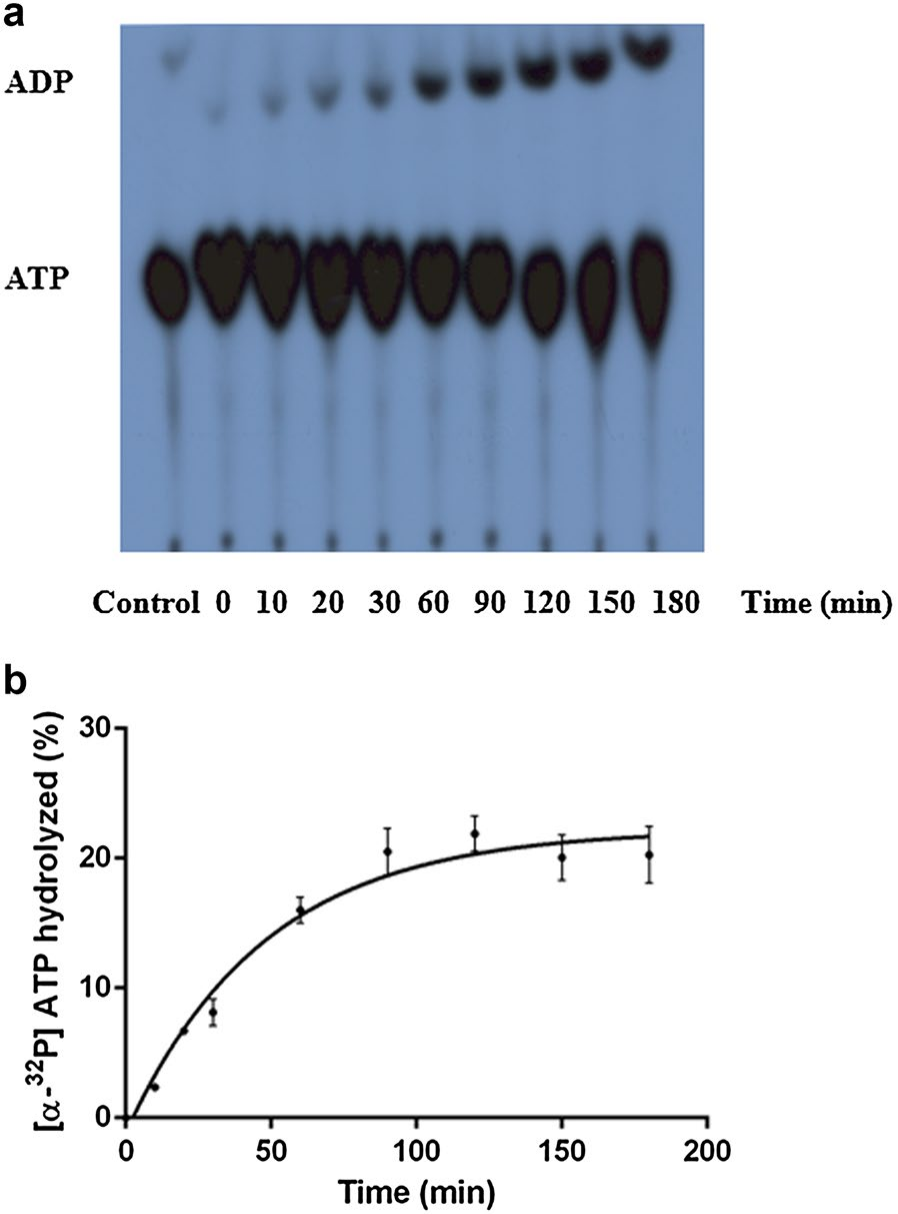
Time-dependent ATPase activity of recombinant PfRuvB3. ATPase activity of PfRuvB3 were assayed using single-stranded DNA (ssM13 mp18) under conditions as described in “PfRuvB3 ATPase activity assay”. ATPase assay was carried out at 37 °C under various incubation time ranging from 10 to 180 min. Control is ATPase assay without enzyme at 37 °C 180 min. **a** Autoradiogram. **b** Kinetics of ATPase activity of recombinant PfRuvB3. Result is shown as mean ± SD of three replicates

The ability of the enzyme to hydrolyze ATP was tested. Recombinant PfRuvB3 revealed a time-dependent ATPase activity in the presence of Mg^2+^ when either single- or double-stranded DNA was added to the reaction mixture (Fig. 5).

### Effect of DNA helicase inhibitors on PfRuvB3 activity

**Fig. 6:**
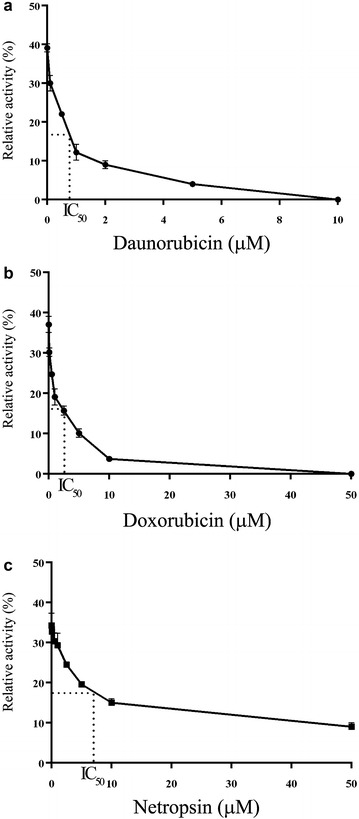
Effects of helicase inhibitors on unwinding activity of PfRuvB3. PfRuvB3 activities were investigated under the assay condition with the presence of various concentrations of daunorubicin (**a**), doxorubicin (**b**) and netropsin (**c**)

**Fig. 7:**
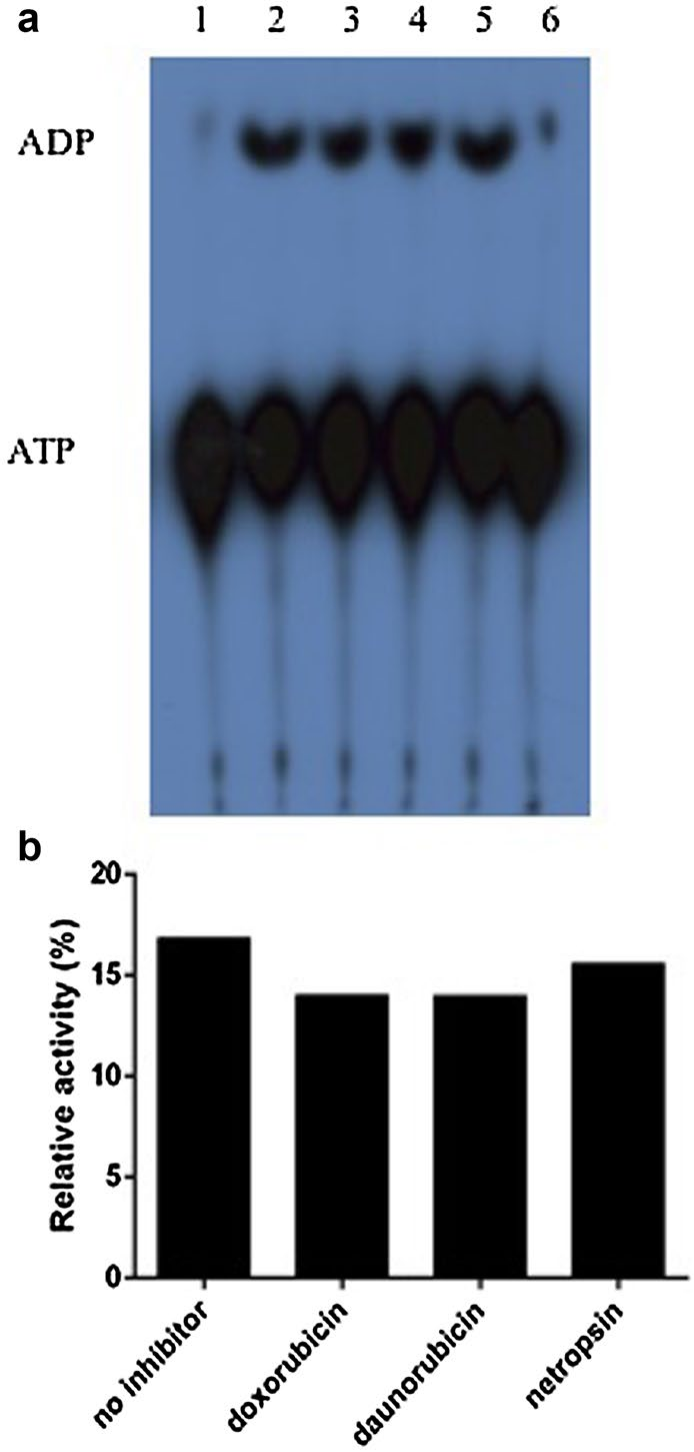
Effects of helicase inhibitors on the ATPase activity of PfRuvB3. **a** Autoradiography of the thin layer chromatography plate. The negative control reactions were performed under standard condition without enzyme (*Lane 1*). *Lane 2* is the assay reaction with enzyme, and *lanes 3*–*5* are ATPase activity of PfRuvB3 in the presence of 50 μM of doxorubicin, daunorubicin and netropsin respectively. *Lane 6* is the assay reaction with heat-inactivated enzyme. **b** quantification of the ATP hydrolysis in an absence and the presence of inhibitors

Known DNA helicase inhibitors such as daunorubicin and doxorubicin, DNA intercalators and netropsin, a minor groove binder were potent inhibitors of PfRuvB3 helicase activity with IC_50_ value of 0.76, 2.6 and 7.09 μM, respectively (Fig. 6), but not aphidicolin, a DNA polymerase inhibitor; genistein, a tyrosine kinase inhibitor and mitoxantrone, a DNA intercalator (IC_50_ value >50 μM). However, three effective helicase inhibitors showed very low inhibition on PfRuvB3 ATPase activity at a concentration of 50 μM (Fig. 7).

## Discussion

RuvBs are highly conserved ATP-binding proteins that belong to the AAA+ family of ATPases that are present in many organisms and are implicated in many cellular pathways [13, 14, 23–25]. In the *P. falciparum* genome, three *P. falciparum* proteins have been annotated as putative RuvB proteins. Phylogenetic comparison of the three PfRuvB protein sequences with those of *E. coli*, yeast and humans reveals that PfRuvB1 and PfRuvB2 are closer to human/yeast RuvBL1, but PfRuvB3 appears to be a homolog of human/yeast RuvBL2 [19].

A previous study [20] reported that PfRuvB3 isolated directly from the parasite organism displayed both DNA helicase and ATPase and activities, but the recombinant protein is able only to recapitulate the ATPase activity [20]. Because the recombinant protein in the previous study originated from resolubilized bacteria-expressed inclusion bodies, it is possible that the refolded PfRuvB3 lost the requisite motif(s) to unwind DNA duplexes.

With that in mind, full-length *PfRuvB3* of *P. falciparum* K1 strain was cloned using pQE-TriSystem His-Strep 2 Vector expression vector whereby the *Strep*-tag sequence (for identification in western blotting) is fused to PfRuvB3 N-terminus and the 8x His-tag sequence (for affinity purification) is located at the C-terminus, so as to minimize misfolding of the resulting recombinant protein. DNA sequencing revealed that *RuvB3* of *P. falciparum* K1 strain is 100% identical to that of strain 3D7 (NCBI database accession no. XM001350297.1). This putative PfRuvB3 (54 kDa) was confirmed by western blot and mass spectral analysis.

Purified heterologously expressed soluble recombinant PfRuvB3 has ATP-dependent DNA helicase without the need for any protein partner, a property similar to that of human RuvBL2 [26]. However, PfRuvB2/PfRuvB3 complex exhibits a higher helicase activity than PfRuvB2 alone [19]. Electron microscopic images of yeast Rvb1/Rvb2 complex showed conformational changes after exposure to nucleotide [27–29] and this complex has stronger DNA helicase activity than the individual proteins [28]. On the other hand, human RuvBL1/RuvBL2 complex has ATPase activity but lacks helicase function [27]. It is possible that in a complex form the enzyme(s) may require additional cofactors for appreciable helicase activity or that to become fully active the enzyme complex needs to establish a tight-binding ATP pocket. Thus, whether a DNA helicase functions better alone or as a complex depends on the species of interest.

Recombinant PfRuvB3 unwinds DNA duplexes containing short (17-mer), long (34-mer) and fork-like (32 and 47-mer) oligonucleotides similar to *Mycoplasma pneumoniae* RuvB homolog [30]. That PfRuvB3 is unable to handle blunt-ended duplex DNA substrate indicates that the enzyme requires a stretch of single-stranded DNA for initial binding, a property similar to that of *E. coli* RuvA [31].

Recombinant PfRuvB3 is dependent on the presence of divalent cations and ATP as found in other organisms [25–27, 30, 31]. High concentrations of divalent cations and salt were suggested to alter protein conformation or to interact directly to negatively charged groups of the enzyme, resulting in decreased enzyme activity [32, 33]. Approximately, 98% of enzyme activity was inhibited by NaCl at concentration of 200 mM (Table 2) which is higher than 154 mM of the physiological concentration. Interestingly, optimal PfRuvB3 helicase activity is able of utilizing dATP (but not the other three dNTPs), a feature it shares in common with that of *E. coli*, *M. pneumonia*, yeast and human homologs [30, 34, 35], although bacterial homologs show a relatively low level response in the presence of dCTP [35, 36]. These results suggest that ATP and dATP may promote stable formation of the conformation required for interaction between NTP and DNA binding domain of PfRuvB3. Similar to homologs in *E. coli*, *Mycoplasma pneumonia*, *Mycoplasma genitalium*, yeast and humans [25, 27, 36–38], recombinant PfRuvB3 manifests ATPase activity in the presence of single- and double-stranded DNA.

Recombinant PfRuvB3 helicase activity was susceptible to inhibition by some DNA intercalators (daunorubicin and doxorubicin) but not to others (genistein and mitoxantrone), to DNA minor groove binder netropsin and not to non-intercalating topoisomerase inhibitor aphidicolin. None of these six compounds affected PfRuvB ATPase activity. All the effective inhibitors bind strongly to DNA [39], and so they most likely interfere with PfRuvB3 unwinding function, whose domain ought to be distant from that of ATPase. PfRuvB3 is more sensitive to daunorubicin (IC_50_ of 0.76 μM) than other helicases, such as *P. falciparum* DNA helicase A (PfDH A) [22], *P. falciparum* UvrD helicase (PfUDN) [40], *P. falciparum* 45 kDa helicase (PfH45) [41], *P. falciparum* 60 kDa DNA helicase (PfDH60) [42] and human DNA helicase (HDH II) [43] (IC_50s_ of 2.0, 4.4, 5.0 3.0, 6.23 μM, respectively). However, it is less sensitive to netropsin (IC_50_ of 7.09 μM) than PfUDN, PfH45, PfDH60 and *P. falciparum* Dbp5/DDX19 homolog (PfD66) [44] (IC_50s_ of 3.3, 0.8, 0.5, 3.2 μM, respectively). Inhibition of PfRuvB3 activity by daunorubicin (IC_50_ of 0.76 μM) and doxorubicin (IC_50_ of 2.6 μM) did not correlate with parasite growth inhibition (IC_50s_ of 2.5 and 1.5 μM respectively) [22] suggesting that they may have different cell permeability and metabolic properties.

## Conclusions

The availability of heterologously expressed soluble recombinant PfRuvB3 has permitted characterization of both its helicase and ATPase properties. Given the differences between malaria parasite and host RuvB in size, amino acid sequence and sensitivity to known helicase inhibitors, such as daunorubicin, recombinant PfRuvB3 may provide an opportunity for further design of novel and more specific inhibitors in the future.

## References

[1] WHO. World Malaria report 2015. Geneva, World Health Organization. 2015.

[2] Ashley EA, Dhorda M, Fairhurst RM, Amaratunga C, Lim P, Suon S (2014). Spread of artemisinin resistance in *Plasmodium falciparum* malaria. N Engl J Med.

[3] Sahu NK, Sahu S, Kohli DV (2008). Novel molecular targets for antimalarial drug development. Chem Biol Drug Des.

[4] Lohman TM (1992). *Escherichia coli* DNA helicases: mechanisms of DNA unwinding. Mol Microbiol.

[5] Tuteja N (2003). Plant DNA helicases: the long unwinding road. J Exp Bot.

[6] Privezentzev CV, Keeley A, Sigala B, Tsaneva IR (2005). The role of RuvA octamerization for RuvAB function in vitro and in vivo. J Biol Chem.

[7] Baharoglu Z, Petranovic M, Flores MJ, Michel B (2006). RuvAB is essential for replication forks reversal in certain replication mutants. EMBO J.

[8] Mezard C, George H, Davies AA, van Gool AJ, Zerbib D, Stasiak A (1999). *Escherichia coli* RuvBL268S: a mutant RuvB protein that exhibits wild-type activities in vitro but confers a UV-sensitive ruv phenotype in vivo. Nucleic Acids Res.

[9] He AS, Rohatgi PR, Hersh MN, Rosenberg SM (2006). Roles of *E. coli* double-strand-break-repair proteins in stress-induced mutation. DNA Repair.

[10] Lim CR, Kimata Y, Ohdate H, Kokubo T, Kikuchi N, Horigome T (2000). The *Saccharomyces cerevisiae* RuvB-like protein, Tih2p, is required for cell cycle progression and RNA polymerase II-directed transcription. J Biol Chem.

[11] ZaO Jónsson, Dhar SK, Narlikar GJ, Auty R, Wagle N, Pellman D (2001). Rvb1p and Rvb2p Are Essential components of a chromatin remodeling complex that regulates transcription of over 5 % of yeast genes. J Biol Chem.

[12] Ohdate H, Lim CR, Kokubo T, Matsubara K, Kimata Y, Kohno K (2003). Impairment of the DNA binding activity of the TATA-binding protein renders the transcriptional function of Rvb2p/Tih2p, the yeast RuvB-like protein, essential for cell growth. J Biol Chem.

[13] Qiu XB, Lin YL, Thome KC, Pian P, Schlegel BP, Weremowicz S (1998). An eukaryotic RuvB-like protein (RUVBL1) essential for growth. J Biol Chem.

[14] Bauer A, Huber O, Kemler R (1998). Pontin52, an interaction partner of beta-catenin, binds to the TATA box binding protein. Proc Natl Acad Sci USA.

[15] Matias PM, Gorynia S, Donner P, Carrondo MA (2006). Crystal structure of the human AAA+ protein RuvBL1. J Biol Chem.

[16] Gangwar D, Kalita MK, Gupta D, Chauhan VS, Mohmmed A (2009). A systematic classification of *Plasmodium falciparum* P-loop NTPases: structural and functional correlation. Malar J..

[17] Ahmad M, Tuteja R (2012). *Plasmodium falciparum* RuvB proteins: emerging importance and expectations beyond cell cycle progression. Commun Integr Biol..

[18] Ahmad M, Tuteja R (2013). *Plasmodium falciparum* RuvB1 is an active DNA helicase and translocates in the 5′-3′ direction. Gene.

[19] Ahmad M, Tuteja R (2013). *Plasmodium falciparum* RuvB2 translocates in 5′-3′ direction, relocalizes during schizont stage and its enzymatic activities are up regulated by RuvB3 of the same complex. Biochim Biophys Acta.

[20] Ahmad M, Singh S, Afrin F, Tuteja R (2012). Novel RuvB nuclear ATPase is specific to intraerythrocytic mitosis during schizogony of *Plasmodium falciparum*. Mol Biochem Parasitol.

[21] Thaithong S, Beale GH, Chutmongkonkul M (1983). Susceptibility of *Plasmodium falciparum* to five drugs: an in vitro study of isolates mainly from Thailand. Trans R Soc Trop Med Hyg.

[22] Suntornthiticharoen P, Petmitr S, Chavalitshewinkoon-Petmitr P (2006). Purification and characterization of a novel 3′-5′ DNA helicase from *Plasmodium falciparum* and its sensitivity to anthracycline antibiotics. Parasitol..

[23] Salzer U, Kubicek M, Prohaska R (1999). Isolation, molecular characterization, and tissue-specific expression of ECP-51 and ECP-54 (TIP49), two homologous, interacting erythroid cytosolic proteins. Biochim Biophys Acta.

[24] Wood MA, McMahon SB, Cole MD (2000). An ATPase/helicase complex is an essential cofactor for oncogenic transformation by c-Myc. Mol Cell.

[25] Tsaneva IR, Müller B, West SC (1993). RuvA and RuvB proteins of *Escherichia coli* exhibit DNA helicase activity in vitro. Proc Natl Acad Sci USA.

[26] Kanemaki M, Kurokawa Y, Matsu-ura T, Makino Y, Masani A, Okazaki K (1999). TIP49b, a new RuvB-like DNA helicase, is included in a complex together with another RuvB-like DNA helicase, TIP49a. J Biol Chem.

[27] Puri T, Wendler P, Sigala B, Saibil H, Tsaneva IR (2007). Dodecameric structure and ATPase activity of the human TIP48/TIP49 complex. J Mol Biol.

[28] Gribun A, Cheung KLY, Huen J, Ortega J, Houry WA (2008). Yeast Rvb1 and Rvb2 are ATP-dependent DNA helicases that form a heterohexameric complex. J Mol Biol.

[29] Torreira E, Jha S, Lopez-Blanco JR, Arias-Palomo E, Chacon P, Canas C (2008). Architecture of the pontin/reptin complex, essential in the assembly of several macromolecular complexes. Structure..

[30] Estevao S, Sluijter M, Hartwig NG, van Rossum AMC, Vink C (2011). Functional Characterization of the RuvB Homologs from *Mycoplasma pneumoniae* and *Mycoplasma genitalium*. J Bacteriol.

[31] Tsaneva IR, West SC (1994). Targeted versus non-targeted DNA helicase activity of the RuvA and RuvB proteins of *Escherichia coli*. J Biol Chem.

[32] Akhtar MS, Ahmad A, Bhakuni V (2002). Divalent cation induced changes in structural properties of the dimeric enzyme glucose oxidase: dual effect of dimer stabilization and dissociation with loss of cooperative interactions in enzyme monomer. Biochemistry.

[33] Warren JC, Cheatum SG (1966). Effect of neutral salts on enzyme activity and structure. Biochemistry.

[34] Kanemaki M, Kurokawa Y, Matsu-ura T, Makino Y, Masani A, K-i Okazaki (1999). TIP49b, a new RuvB-like DNA helicase, is included in a complex together with another RuvB-like DNA helicase, TIP49a. J Biol Chem.

[35] Parsons CA, West SC (1993). Formation of a RuvAB-Holliday junction complex in vitro. J Mol Biol.

[36] Estevao S, Sluijter M, Hartwig NG, van Rossum AM, Vink C (2011). Functional characterization of the RuvB homologs from *Mycoplasma pneumoniae* and *Mycoplasma genitalium*. J Bacteriol.

[37] Jha S, Dutta A (2009). RVB1/RVB2: running rings around molecular biology. Mol Cell.

[38] Huen J, Kakihara Y, Ugwu F, Cheung KL, Ortega J, Houry WA (2010). Rvb1-Rvb2: essential ATP-dependent helicases for critical complexes. Biochem Cell Biol.

[39] Marco A, Arcamone F (1975). DNA complexing antibiotics: daunomycin, adriamycin and their derivatives. Arzneimittelforschung.

[40] Tarique M, Tabassum F, Ahmad M, Tuteja R (2014). Malaria Group. *Plasmodium falciparum* UvrD activities are downregulated by DNA-interacting compounds and its dsRNA inhibits malaria parasite growth. BMC Biochem.

[41] Pradhan A, Tuteja R (2007). Bipolar, Dual *Plasmodium falciparum* helicase 45 expressed in the intraerythrocytic developmental cycle is required for parasite growth. J Mol Biol.

[42] Pradhan A, Tuteja R (2006). *Plasmodium falciparum* DNA helicase 60. dsRNA- and antibody-mediated inhibition of malaria parasite growth and downregulation of its enzyme activities by DNA-interacting compounds. FEBS J.

[43] Bachur NR, Yu F, Johnson R, Hickey R, Wu Y, Malkas L (1992). Helicase inhibition by anthracycline anticancer agents. Mol Pharmacol.

[44] Mehta J, Tuteja R (2011). Inhibition of unwinding and ATPase activities of *Plasmodium falciparum* Dbp5/DDX19 homolog. Commun Integr Biol..

